# Unclean Mouth and Its Evil Results*Read in the Section on Stomatology of the American Medical Association, at the Sixty-Third Annual Session, held at Atlantic City, June 1912.

**Published:** 1912-08-15

**Authors:** M. H. Fletcher

**Affiliations:** Cincinnati


### UNCLEAN MOUTH AND ITS EVIL RESULTS.*

M. H. FLETCHER, D.D.S., M.D., M.S., CINCINNATI.

Uncleanliness of the mouth is probably the indirect cause of more systemic diseases than any other thing.

"Chronic ulcerative stomatitis," "putrid sore mouth," "fetid sore mouth," etc., as described in works on med-

*Read in the Section on Stomatology of the American Medical Association, at the Sixty-Third Annual Session, held at Atlantic City, June 1912.

icine, and as spoken of by physicians, are simply stages of a disease of the alveolar process and gums, popularly known as "receding gums," "sore," "spongy," or "bleeding gums," conditions which are preventable by proper daily cleanliness at the hands of the patient. They are caused by local irritants of the gums, calcareous deposits and other irritants, such as bands, rough edges of cavities and roots of teeth, rough fillings, splinters, or improper approximal spaces may be the exciting cause.

After the onset or initial lesion in the gums, and the alveolar process has become involved, the malady is primarily that of the bone, the soft, spongy and bleeding gums being a natural sequence. This is evidenced by the fact that the gum-tissues, although still inflamed, remain intact and nearly to their normal height long after deep pockets have been formed into the bone. The gums also promptly recover their normal health when the bone gets well or the tooth is removed. Again no amount of gum treatment, pure and simple, will cure the disease.

The disease may be described in all its phases by arbitrarily dividing it into stages, as follows:

Initial or simple alveolitis.

Non-supperative alveolitis.

Suppurative alveolitis.

Necrotic alveolitis.

Acute alveolitis.

Descriptive subdivisions are:

Chronic non-suppurative alveolitis.

Chronic suppurative alveolitis.

Necrotic non-suppurative alveolitis, which is always chronic.

Necrotic suppurative alveolitis, which is nearly always chronic but may be acute.

The subdivisions are merely desriptive of combined conditions. The suppurative and necrotic stages, both acute and chronic, display all the conditions described under the names, "chronic ulcerative stomatitis," "fetid stomatitis,"

"putrid sore mouth," "chronic stomatitis," etc. Insanitary conditions of the mouth undoubtedly encourage the propagation and virility of such microbes as are involved in aphtha, thrush, cancrum oris, and other bacterial diseases of the mucous membrane.

Gingivitis is always present to some degree in every stage of alveolitis and is very marked in the advanced stages. Its mildness or intensity is dependent on the condition of the underlying bone, the character of the infection and the quantity and character of the calculous about the teeth. Mercury and other metallic poisonings intensify the symptoms as do auto-intoxications, scurvy and sordes.

### INITIAL ALVEOLITIS.

Initial alveolitis means the beginning of the disease in the gum-margin at the necks of th teeth. It usually starts from local irritants. These irritants cause wounds in the gum-tissues, which wounds are the doors of entrace (the infection atrium) of the infectious bacteria. All the irritants mentioned, excepting tartar, simply create the wound, which may be perpetuated by microbes. Tartar, however, never ceases to be deposited and is a continuous and progressive irritant, perpetuating the disease in its simple form.

### CHRONIC NON-SUPPURATIVE ALVEOLITIS.

The unremitting effort of the tissues to ward off disease may for years prevent a malignant infection of these wounds. With some persons it may be for a life-time, but the ultimate end of the disease, if the patient lives long enough and the deposits are not removed, is the loss of the tooth.

The simple or chronic non-suppurative form is so-called simply because the pus is not visible and is the most common of all forms. When, however, a malignant type of infection finds encouraging environment in one of these chronic non-suppurative wounds, it is most apt to be suppurative in type. This is much more rapid in its destructive progress than the non-suppurative form. When suppuration exists in one place it is apt to be found in several places, and many times it is found in all the sockets. This condition constitutes suppurative alveolitis or so-called pyorrhea alveolaris.

### SUPPURATIVE ALVEOLITIS OR PYORRHEA ALVEOLARIS.

When pus is exuding from the sockets, it may be in such quantities that it is easily seen, or it may be easily pressed out with the fingers. On the other hand, it may be in such small quantities that it can only be seen with the aid of the microscope.

The absence of deposits on the roots of some of these teeth has led many authors to say that calculus or other local irritants have little or nothing to do with the disease. They say that it is systemic in its origin, that it is an accompaniment of an uric acid diathesis or the result of arteriosclerosis and its accompaniment. If, however, the complete history of alveolitis could be known, I believe, without doubt, that it would be found that the disease always starts with a local irritant and lesion. It is perpetuated by the continued presence of calculus, or of calculus and infection, or by other local irritants and infection, as above described. Those who say that the disease is caused by other than local irritants admit that these irritants must be thoroughly removed, and the pockets sterilized, or the teeth removed, before the disease will disappear. The tartar or other irritants at the necks, which caused the initial lesion, may have been removed and thus unobserved. The tissues, however, may still be suppurative. It must be remembered that there can be no lesion without a cause, and no infection without a lesion.

### NECROTIC ALVEOLITIS.

When the cancellous bone about or beyond the roots is reached any germs capable of living in such environment not only find less resistance, but greater protection and more favorable conditions for rapid multiplication; consequently, considerable zones of bone die. This necrotic bone becomes an additional object of care to the elements of protection and repair. In this stage of the disease, calculus is often found about the apices, especially of the upper molars and the antrum is frequently penetrated, which often means empyema of this cavity.

Deep-seated necrosis in the lower jaw sometimes result in the necessity of removing quite a portion of either the outer or inner plate of the jaw, and the loss of several teeth.

In some instances large necrotic zones are found in the bone beyond the teeth. These cavities are often not associated with pus, but are of a "dry" necrotic or carious character and are not accessible without drilling or curetting into them. The symptoms leading to their existence are usually pain of a neuralgic or tic douloureux character. The treatment given in these instances is usually systemic for neuritis, instead of surgical for diseased periosteum and bone. Consequently the patient often suffers for nany years without relief. Removal of the teeth involved gives entire relief in time. Healing may be induced, however, by curetting or burring out the diseased bone, sterilizing the cavities, and persisting in the treatment until recovery takes place.

MANUAL TREATMENT.

While there is probably no disease of mankind so prevalent as alveolitis, there is likely no disease so amenable to treatment. The treatment is largely local and manual. There is no disease more surely preventable; it is a question of keeping the mouth clean and the teeth free fro n tartar and other irritants about their necks. This is largely a matter of mechanical cleaning and not medicinal. If the cause of the lesions is prevented, infection cannot enter and sterilizing medicines are not needed. Many dentists are now engaged in the successful treatment of alveolitis and in teaching the patients how to prevent it.

The whole of the operative treatment consists in removing all deposits or other irritants from the roots and necks of the teeth. This must be followed by sterilizing these surfaces, removing the dead and diseased bone in and about the sockets, sterilizing the sockets and cavities of bone, and keeping them thus sterilized until recovery is complete.

The preventive treatment is the patient's work. He needs instruction from the physician how to do it, however, since the ordinary way of "scrubbing the teeth" falls far short of reaching the object. In my hands it requires from three to six months for patients properly to learn the process of cleaning. It is the most important feature of the whole subject and the only practical method of prevention. Patients can and do learn it, however, and in consequence have mouths pure and free from disease, which is a large factor of insurance against many systemic maladies. Patients may have to return two or three times a year to have places cleaned which they cannot easily reach. But they can and do learn the preventive treatment.

The greatest shortcoming, in treating the disease, is the lack of thoroughness on the part of the stomatologist. He often fails to remove the deep-seated deposits near the apices of the roots and the dead bone beyond. To do this properly requires a touch trained to distinguish between deposit and the tooth surface, and between live and dead bone. It also requires skill and conscientious effort coupled with a knowledge of the anatomy and pathology. Thus only can the work be properly done. This operative procedure must be followed by suitable methods of sterilizing the wounds. In but few cases it is practicable to remove all dead and diseased bone and all infection. If, however, a sufficient amount be removed, Nature will be able to throw a large enough army of repair about the wound to destroy the remaining bacteria and remove the dead bone. She then rebuilds the destroyed tissue as far as possible.

My own practice, in sterilizing these pockets, is to use pure tincture of iodin, 75 per cent. lactic acid, or crystals of argyrol. Many other remedies may be equally good, but each operator must select his own, keeping in mind the object to be accomplished. A long curved hypodermic needle, and cotton wound on a "twist broach" have in my hands permitted the medication of any case yet presented. I have devised and had manufactured a set of curets and engine burs for removing dead and diseased bone. The burs are usually more efficient and less painful. The "plane-bit" type of the cleaners, however, is sufficient in most cases, being used also as curets.

PREVENTIVE TREATMENT.

The dental profession of to-day is doing great good in promulgating and teaching the value of oral hygiene. After mouth examinations in schools, lectures and demonstrations are given to the children, teachers and mothers on the prevention of disease by mouth cleanliness. The results of this work show, not only improvement in general health, but also greatly increased mental capacity in the children.

The condition of the mouth, in both adults and children, should receive much more careful attention from surgeons and physicians than it ever has. This is especially needful, since oral sepsis is now known to be the source of many diseases. Numbers of these are surely preventable by mouth cleanliness. Insurance companies are beginning to recognize this fact.

The so-called prophylactic treatment, as practiced by the dentists of to-day, is really the surgical treatment of the disease, since it is the removal of the exciting and secondary causes.

In the initial and non-suppurative stages of alveolitis, simply removing the tartar—cleaning the teeth—enables the wounds to heal. This is all the treatment a large majority of cases receive at the hands of the most dentists, and all they need. But it is most desirable to know the whole pathology and treatment of the extreme cases as well as the simple ones.

The disease tends strongly to return if preventive treatment is neglected by the patient. That which brought on the disease, namely, the lime salts in the saliva, is always present to bring it back. Tartar, when first deposited, is soft, like partly dissolved soap, and is easily removed. Inside of forty-eight hours the film next to the tooth begins to harden and is then removed with much difficulty. Hence comes the necessity of thoroughly cleaning the mouth and teeth at least once a day, with brush, powder, picks and floss.

### RECOVERY.

It should be borne in mind that, after the initial stage, alveolitis is a bone disease, and should be treated as such. It takes the bone and periosteum of the jaws as long to repair as it does like tissues elsewhere; the cementum requires a longer time for repair than does bone.

In the deep-seated cases the difficulties of removing all calculus and infection are such that a number of sittings, a week or two apart, may be necessary to remove them. Healing begins immediately after the first treatment. The usual time required for regeneration of the bone and periosteum, however, must be allowed before we can expect recovery. Therefore great patience is necessary on the part of both patient and operator. Three months is the shortest time in which one could reasonably expect good results. A period of six months, a year or more often elapses before recovery is satisfactory. During this time the patient will need professional assistance once a week, or two or more weeks apart, according to symptoms.

### SYSTEMIC FEATURES.

Alveolitis, like all other diseases, has its nervous phase.

This results in discomfort, pain, or total disability, according to the complications and intensity of the disease.

Alveolitis is encouraged by auto-intoxications and by the numerous manifestations accompanying them, such as gout, uric acid disorders, arteriosclerosis, etc. These diseases are in turn encouraged by alveolitis; thus, the vicious circle is established. I know of no systemic pathologic conditions, however, which could cause the almost universal presence of alveolitis, but local irritants are universally present.

We all know that alveolitis recovers without systemic treatment, but such treatment often materially assists in recovery. During systemic disorders, any weakened organ or point of least resistance, such as "sore gums," becomes easily inflamed and acute. That particular local feature is thus intensified. If, therefore, systemic disorders are manifest and serious, they should have attention at the hands of the operator or the family physician. On the other hand, I believe that alveolitis not only is of local origin, but also, in its suppurative and necrotic stages, is a great predisposing factor to, and is the cause of, many bodily diseases, such as pernicious anemia, disorders of the alimentary tract, nervous derangement, etc., etc. The mouth as a source of septic poisoning and the cause of many diseases has recently attracted the attention of acute diagnosticians.—Abstract of paper published in *Journal A. M. A.*, July 13, 1912.

